# Data on microstructural and optoelectronic properties of electrodeposited silver mesh transparent conducting electrodes

**DOI:** 10.1016/j.dib.2023.109238

**Published:** 2023-05-18

**Authors:** Seoin Kang, Choong-Heui Chung

**Affiliations:** a Department of Materials and Manufacturing Engineering, Hanbat National University, Daejeon, 34158, Republic of Korea; b Department of Materials Science and Engineering, Hanbat National University, Daejeon, 34158, Republic of Korea

**Keywords:** Transparent conducting electrode, Metal mesh, Electrodeposition, Self-cracking template

## Abstract

Electrodeposited Ag mesh transparent conducting electrodes (TCEs) based on self-cracking templates can achieve high optical transmittances and low sheet resistances by controlling the shape of the self-cracking templates and electrodeposition duration. The surface coverage of the mesh is mainly determined by the surface shape of the self-cracking template. Electrodeposition of Ag can adjust the thickness of the mesh, significantly reducing the sheet resistance while maintaining the high optical transmittance of the TCEs. The TCE electrodeposited for 30 s exhibited an optical transmittance as high as 88.4% and a sheet resistance as low as 2.24 Ω/□. Here we provide the microstructural and optoelectronic performance data of the electrodeposited Ag mesh TCEs.

**Specifications Table**

**Table:** 

Subject	Materials Science
Specific subject area	Transparent conducting electrodes
Type of data	Images and Figures
How the data were acquired	The optical transmittance of the Ag mesh was measured by a UV–Vis spectrometer (GENESYS 10S Vis, SCINCO) using a glass substrate as a reference. The sheet resistance of the Ag mesh was measured by a 4-point probe (FPP-40 K, DASOL ENG). Microstructural data of the Ag mesh were obtained using a scanning electron microscope (SEM) (FE-SEM, HITACHI SU8230).
Data format	Raw and analyzed
Description of data collection	The microstructural and optoelectronic data of the Ag mesh were obtained as a function of electrodeposition time of Ag. SEM images were taken at acceleration voltage of 5 kV at working distance of 5- 10 mm. The optical transmittance and the sheet resistance of samples were measure in an air atmosphere at room temperature
Data source location	Hanbat National University, Daejeon 34158, Republic of Korea
Data accessibility	Repository name: Mendeley Data Data identification number: 10.17632/3k5y2jcpk5.1 Direct URL to data: https://data.mendeley.com/datasets/3k5y2jcpk5/1
Related research article	Kang, S., Arepalli, V.K., Yang, E. et al. High Performance and Flexible Electrodeposited Silver Mesh Transparent Conducting Electrodes Based on a Self-Cracking Template. Electron. Mater. Lett. **18**, 440–446 (2022). https://doi.org/10.1007/s13391-022-00358-4

## Value of the Data

• Metal mesh TCEs have emerged as very promising alternatives to ITO in next-generation optoelectronic devices. Our electrodeposited Ag mesh possesses favorable characteristics, such as a low sheet resistance of 2.24 Ω/□ and high optical transmittance of 88.4%, making it a suitable choice for various optoelectronic devices. Moreover, electrodeposition offers a cost-effective alternative to other techniques like lithography or vacuum deposition.
• The authors recently reported the improved mechanical and optoelectronic performances of Ag mesh transparent conducting electrodes (TCEs) prepared by electrodeposition [1]. Microstructural and optoelectronic data of electrodeposited Ag mesh TCEs are useful for engineers working in the of solar cells, displays, and touch panels which require TCEs.
• The size and shape of the Ag mesh can be adjusted to meet specific application requirements, providing greater flexibility and precision in design. Moreover, electrodeposited Ag mesh TCEs can be deposited on various substrates, including plastic and glass, adding to their versatility and potential for use in a wide range of applications.

## 1 Objective

The metal mesh TCEs have been studied as substitutes for existing transparent conductive materials such as Indium tin oxide (ITO), which are fragile, expensive, and have limited utility. Metal mesh TCEs offer several advantages over ITO in term of mechanical flexibility and optoelectronic properties. The performance of metal mesh TCEs depends on several factors such as wire width, wire thickness, inter-wire spacing, and the material properties of the metal used. Researchers continue to explore ways to optimize the design and manufacturing method of metal mesh TCEs to improve their performance and broaden their application in various industries. Therefore, in this article, we provide data on electrodeposited Ag mesh TCEs based on self-cracking templates. The data obtained can be utilized to tailor the optoelectronic characteristics of individual mesh TCEs, catering to their specific application requirements, thus rendering them suitable for specialized applications.

## 2 Data Description

All raw data presented in this article can be found in a data repository [2]. All data files are in a single folder, where all the figures are saved in jpg format and the raw data presented in the figures are saved in Microsoft Excel format.

In this work, Ag mesh TCEs were fabricated through a two-step process: Step 1. Thermal evaporation of Ag on a self-cracking template. Step 2. Electrodeposition of Ag on the thermal evaporated Ag mesh.

**Fig. 1:**
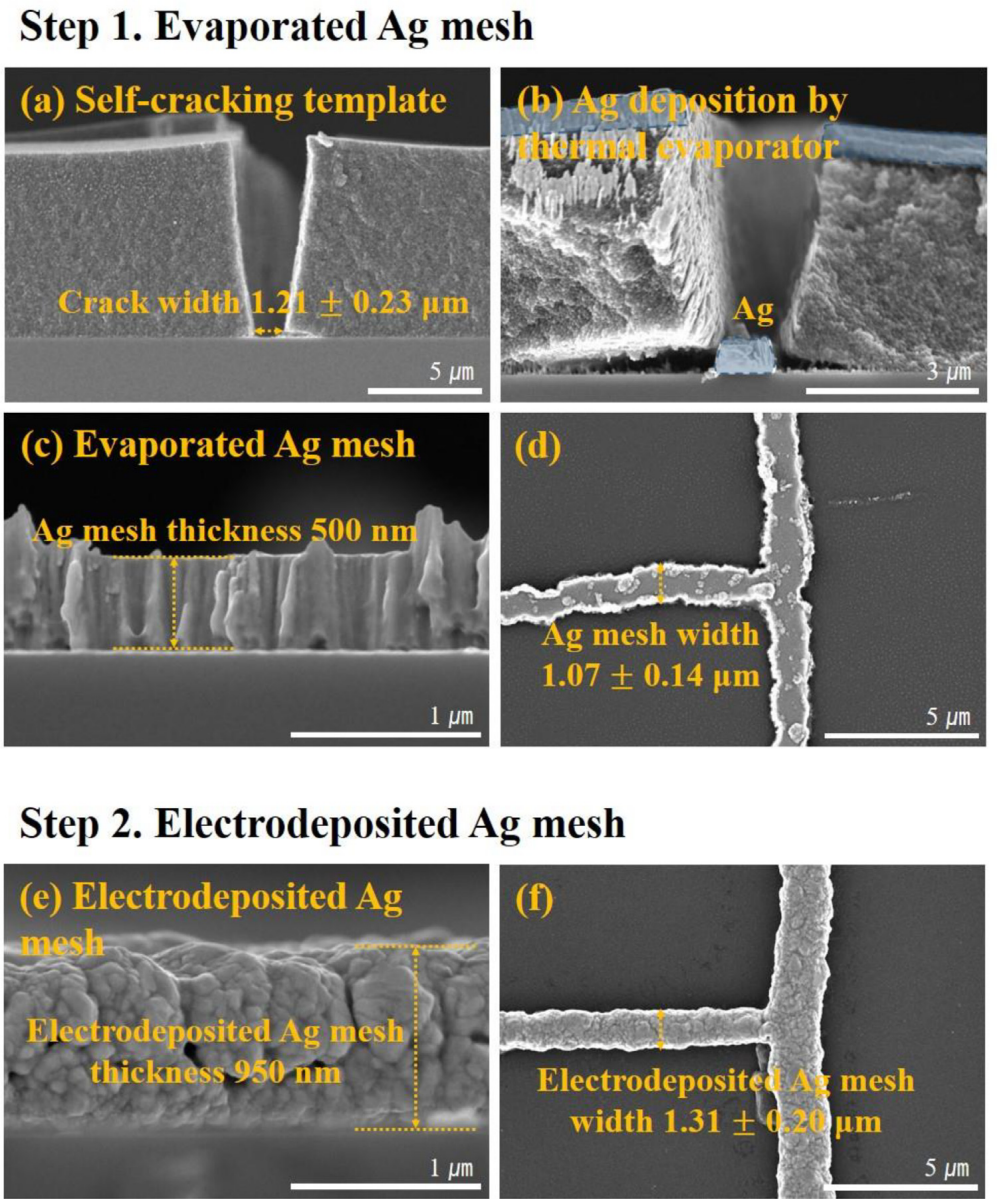
Scanning Electron Microscope (SEM) image showing the microstructure at each stage. (a) cross-sectional view of a self-cracking template formed by spin coating, (b) cross-sectional view of a thermal evaporated Ag formed onto the template, (c) cross-sectional and (d) plane view of the resulting thermal evaporated Ag mesh after removing the template, (e) cross-sectional and (f) plane view of the electrodeposited Ag mesh.

The microstructures of a sample in each step are shown in Fig. 1. Self-cracking templates obtained by spin coating the self-cracking solution are U-shaped (Fig. 1(a)). Fig. 1(b) shows the outcome of thermal evaporated Ag onto the self-cracking template. U-shaped geometry of the crack allows for thermal evaporated Ag to be deposited even on the sidewalls, thereby restricting the maximum thickness of the evaporated Ag where the self-cracking template can be successfully lifted off. Thus, it is crucial to deposit thermal evaporated Ag with an appropriate thickness that would not interfere with the mesh formation process. The thermal evaporated Ag mesh is formed by lifting the template off, as shown in Fig. 1(c, d). The Ag mesh width is evidently reliant on the crack width, which can be regulated by adjusting the spin coating speed during fabrication [2]. Subsequently, the thermally evaporated Ag mesh was subjected to Ag electrodeposition (Fig. 1(e, f)). Electrodeposition of Ag onto the mesh leads to a modest enlargement of its lateral and vertical size.

**Fig. 2:**
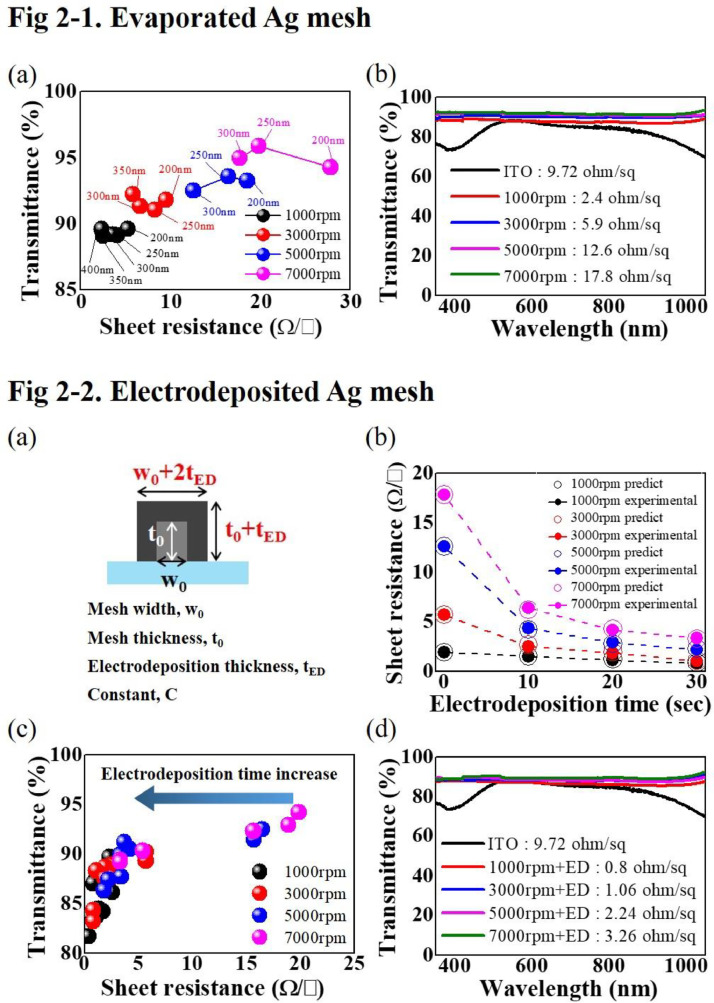
Optoelectronics properties of evaporated Ag meshes, and electrodeposited Ag meshes. 2–1(a) sheet resistance versus optical transmittance at 550 nm of the thermal evaporated Ag meshes with different spin-coating speed of the self-cracking solution and thermal evaporated Ag thickness. 2–1(b) optical transmittance of several thermal evaporated Ag mesh TCEs as a function of wavelength of incident light, 2–2(a) a schematic of the cross-section of the electrodeposited Ag mesh showing the increase of both width and thickness of Ag mesh by electrodeposition. 2–2(b) the measured sheet resistance and predicted sheet resistance using Eq. (1) of the electrodeposited Ag meshes, 2–2(c) the change of the sheet resistance and the transmittance by the electrodeposition, 2–2(d) the optical transmittance of several Ag mesh TCEs, in which Ag was electrodeposited for 30 s.

Fig. 2-1 demonstrates the optoelectronic characteristics of the thermal evaporated Ag meshes. The sheet resistance of the Ag mesh TCE is influenced by both the surface coverage of the mesh and the thickness of the deposited Ag. The surface coverage of the mesh is dictated by the surface shape of the self-cracking template. The increase of the thickness of the thermal evaporated Ag results in the decrease of its sheet resistance, while its transmittance remains almost constant since the surface coverage remains unaltered (Fig. 2-1(a)). Fig. 2-1(b) shows the transmittance of the thermal evaporated Ag mesh TCEs with different sheet resistance. They show the transmittance at 550 nm and the sheet resistance and of 89.2% - 2.4 Ω/□, 91.8% - 5.9 Ω/□, 92.4% - 12.6 Ω/□, and 94.9% - 17.8 Ω/□.

The effectiveness of electrodeposition providing dramatic reduction in the sheet resistance was evaluated (Fig. 2-2) [3], [4]. Electrodeposition process facilitates an increase in both the width and thickness of the mesh, enabling the prediction of sheet resistance as a function of electrodeposition thickness (Fig. 2-2(a)). Eq. (1) was used to predict the sheet resistance (R_S,ED_) of the electrodeposited Ag mesh TCEs.

(1) $$R_{S,\text{ED}}\propto \frac{1}{\left(w_{0}+2t_{\text{ED}}\right)\left(t_{0}+t_{\text{ED}}\right)}$$

Here, w_0_, t_0_ and t_ED_, represent the width and the thickness of the thermal evaporated Ag mesh, and the increased thickness by the electrodeposition of Ag, respectively. Data of w_0_ and t_0,_ t_ED_ as a function of time can be found in our previous publication [2]. For electrodeposition time of 0 ∼ 30 s, the measured sheet resistance values of the Ag mesh TCEs are well matched with the predicted sheet resistance values obtained using Eq. (1) (Fig. 2-2(b)). The change in the transmittance and sheet resistance of the Ag mesh with increasing electrodeposition time are shown in Fig. 2-2(c). Fig. 2-2(d) presents the transmittance of the electrodeposited Ag mesh TCEs with different sheet resistance after the electrodeposition of Ag for 30 s. They show the transmittance at 550 nm and the sheet resistance and of 87.0% - 0.8 Ω/□, 88.0% - 1.06 Ω/□, 88.4% - 2.24 Ω/□, and 89.2% - 3.26 Ω/□. The performance of the Ag mesh TCEs are superior to that of a commercialized ITO film.

The Figure of Merit (FoM) is a metric that quantifies the overall performance of a TCE. It is defined as the ratio of sheet resistance to optical transmittance and is calculated by Eq. (2) [5].

(2) $$\text{FoM}\;\left({\left(\Omega /□\right)}^{-1}\right)=\frac{\sigma_{\text{dc}}}{\sigma_{\text{opt}}}=\frac{188.5}{R_{s}\cdot \left(T_{550}^{-1/2}-1\right)}$$

**Fig. 3:**
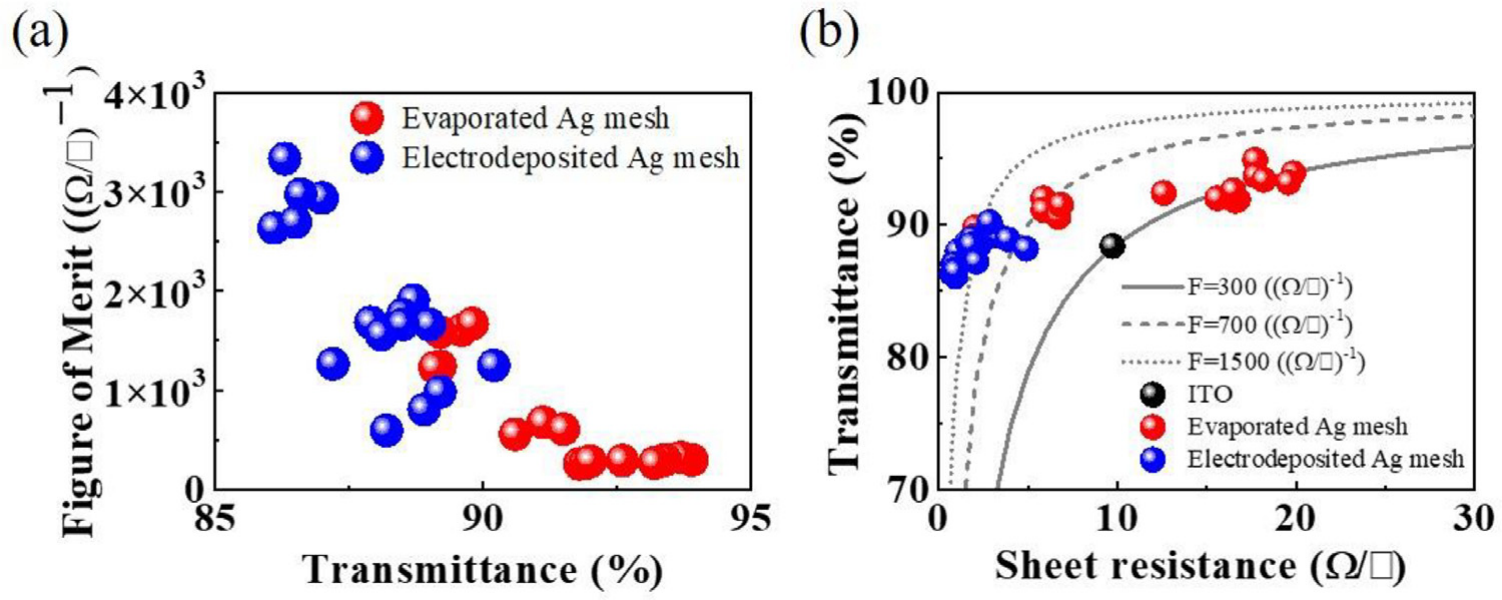
Comparison of the performance of the evaporated Ag meshes and the electrodeposited Ag mesh TCEs. (a) Figure of Merit (FoM) before and after the electrodeposition of Ag, (b) FoM comparison between a commercialized ITO film, the evaporated Ag mesh, and the electrodeposited Ag mesh TCEs.

Higher FoM values indicate a more efficient TCE with lower sheet resistance and higher optical transmittance. The FoM of the commercialized ITO thin film is 300 ${\left(\Omega /□\right)}^{-1}$, the evaporated Ag mesh TCEs showed FoM values in the range of 300 to 700 ${\left(\Omega /□\right)}^{-1}$, and the electrodeposited Ag mesh TCE reached higher FoM values of approximately 1500 ${\left(\Omega /□\right)}^{-1}$, proving the effectiveness of electrodeposition in enhancing the optoelectronic performance of TCEs (Fig. 3(a, b)).

## 3 Experimental Design, Materials and Methods

*Fabrication*: Glass substrates (25 mm × 25 mm × 1 mm) were cleaned using acetone, ethanol, and DI water for 10 min using an ultrasonicator, respectively. CR-735 (Lubrizol), an acrylic resin-based self-cracking solution, was spun on the substrates to form self-cracking templates. After spinning at a rotation speed of 600 rpm for 15–30 s to uniformly spread CR-735 over the whole area of the substrates, the substrates were then rotated at a speed in the range of 1000–7000 rpm for 30 s. The spin-coated substrates were dried at room temperature to obtain self-cracking templates. Then, Ag was deposited using thermal evaporation onto the templates. The thermal evaporated Ag meshes were immersed in acetone for 1 hour and treated using an ultrasonicator for 5 min to remove the templates, which resulted in a thermal evaporated Ag mesh TCEs. Subsequently, Ag electrodeposited was then applied to improve the optoelectronic performance of the mesh TCEs. An Ag electrodeposited solution was prepared by dissolving 7 g of AgCN (Acros Organics, 180230500), 15 g of KCN (Sigma-Aldrich, 207810), 1.5 g of K_2_CO_3_ (Alfa-Aesar, A16625), 5 g of Na_2_O_3_S_2_·5H_2_O (Alfa-Aesar, A17914) in 85 ml of DI water. A thermally deposited Ag mesh TCE substrate and an Ag plate were used as the working and reference electrode, respectively, and immersed in the Ag electrodeposition solution at a constant current source of −4.5 mA.

*Characterization*: The optical transmittance of Ag mesh TCEs were measured using a UV–Visible spectrometer using an employed substrate as a reference. The sheet resistance of Ag mesh TCEs were measured using a 4-point probe. The microstructures of the self-cracking template and Ag mesh TCEs were analyzed using SEM.

## Ethics Statement

Our work meets all ethical requirements for publication in Data in Brief. Our work current work does not involve human subjects, animal experiments, or any data collected from social media platforms.

## CRediT authorship contribution statement

**Seoin Kang:** Investigation, Data curation, Writing – original draft. **Choong-Heui Chung:** Conceptualization, Writing – review & editing, Supervision.

## Declaration of Competing Interest

The authors declare that they have no known competing financial interests or personal relationships that could have appeared to influence the work reported in this paper.

## Data Availability

This dataset contains microstructural and optoelectronic properties of electrodeposited silver mesh transparent conducting electrodes (Original data) (Mendeley Data).

## References

[1] Cho K.S., Kang S., Oh Y.J., Park J.S., Lee S., Wi J.S., Park J.H., Song S., Kim K., Eo Y.J., H.Yun J., Gwak J., Cho J.S., Chung C.-.H. (2022). ACS Appl. Electron. Mater..

[2] Kang S., Chung C.-.H. (2023). Data on microstructural and optoelectronic properties of electrodeposited silver mesh transparent conducting electrodes. Mendeley Data, V1.

[3] Kang S., Arepalli V.K., Yang E., Lee S., Wi J.S., Yun J., Song S., Kim K., Eo Y.J., Cho J.S., Gwak J., Chung Choong-Heui (2022). Electro. Materi. Lett..

[4] Lee S., Jang J., Park T., Park Y.M., Park J.S., Kim Y.K., Lee H.K., Jeon E.C., Lee D.K., Ahn B., Chung C.-.H. (2020). ACS Appl. Mater, Interfaces.

[5] van de Groep J., Spinelli P., Polman A. (2012). Nano Lett..

